# The Interaction Affinity between Vascular Cell Adhesion Molecule-1 (VCAM-1) and Very Late Antigen-4 (VLA-4) Analyzed by Quantitative FRET

**DOI:** 10.1371/journal.pone.0121399

**Published:** 2015-03-20

**Authors:** Sandeep Chakraborty, Shih-Yang Hu, Shu-Han Wu, Artashes Karmenyan, Arthur Chiou

**Affiliations:** 1 Institute of Biophotonics, National Yang-Ming University, Taipei, Taiwan, 11221, ROC; 2 Research Center for Applied Sciences, Academia Scinica, Taipei, Taiwan, 11529, ROC; 3 Department of Physics, National Dong Hwa University, Hualien, Taiwan, 97401, ROC; 4 Biophotonics & Molecular Imaging Research Center, National Yang-Ming University, Taipei, Taiwan, 11221, ROC; University of Oldenburg, GERMANY

## Abstract

Very late antigen-4 (VLA-4), a member of integrin superfamily, interacts with its major counter ligand vascular cell adhesion molecule-1 (VCAM-1) and plays an important role in leukocyte adhesion to vascular endothelium and immunological synapse formation. However, irregular expressions of these proteins may also lead to several autoimmune diseases and metastasis cancer. Thus, quantifying the interaction affinity of the VCAM-1/VLA-4 interaction is of fundamental importance in further understanding the nature of this interaction and drug discovery. In this study, we report an *‘in solution’* steady state organic fluorophore based quantitative fluorescence resonance energy transfer (FRET) assay to quantify this interaction in terms of the dissociation constant (K_d_). We have used, in our FRET assay, the Alexa Fluor 488-VLA-4 conjugate as the donor, and Alexa Fluor 546-VCAM-1 as the acceptor. From the FRET signal analysis, K_d_ of this interaction was determined to be 41.82 ± 2.36 nM. To further confirm our estimation, we have employed surface plasmon resonance (SPR) technique to obtain K_d_ = 39.60 ± 1.78 nM, which is in good agreement with the result obtained by FRET. This is the first reported work which applies organic fluorophore based *‘in solution’* simple quantitative FRET assay to obtain the dissociation constant of the VCAM-1/VLA-4 interaction, and is also the first quantification of this interaction. Moreover, the value of K_d_ can serve as an indicator of abnormal protein-protein interactions; hence, this assay can potentially be further developed into a drug screening platform of VLA-4/VCAM-1 as well as other protein-ligand interactions.

## Introduction

Protein-protein interactions play a critical role in a wide variety of cellular processes. One such interacting protein pair, Vascular Cell Adhesion Molecule-1 (VCAM-1/CD106) and Very Late Antigen-4 (VLA-4), is essential in the interactions of leukocytes with cytokine-activated endothelial cells during transendothelial migrations [1–3] as well as in the formation of immunological synapse [4, 5]. VLA-4 (also known as CD49d/CD29 or α_4_β_1_), a member of integrin superfamily of cell-surface receptors, is a transmembrane non-covalent heterodimer of α_4_ (155 kDa) and β_1_ (150 kDa) subunits, which is expressed on all leukocytes except neutrophils [6–8]. Two major natural ligands of VLA-4 are the alternatively spliced connecting segment (CS-1) of fibronectin [9] and VCAM-1; the latter is a cytokine activated endothelial cell surface receptor protein [10, 11]. VCAM-1 is also a transmembrane adhesion molecule of Ig supergene family and comprising a short cytoplasmic domain, a transmembrane domain, and seven (predominant form) or six extracellular immunoglobulin like domains due to the alternative splicing of mRNA from a single VCAM-1 gene to form two isoforms [12–14].

The affinity of VLA-4 towards VCAM-1 is generally governed by the presence of divalent cations (Mg^2+^, Mn^2+^, and Ca^2+^ etc.) [15]. The I-like domain of the β-subunit forms the head domain in VLA-4 and harbors a metal-ion-dependent-adhesion-site (MIDAS), which binds the divalent cations, as mentioned before, necessary for binding to VCAM-1 [16]. VLA-4 binds to regions within the first (D1) and fourth (D4) domains of the full-length seven domain form of VCAM-1 [17]. A dominant acidic motif QIDSPL within D1 of VCAM-1 is critical for interaction with VLA-4. However, it is interesting to note that binding of VCAM-1 to D4 needs VLA-4 activation (by either divalent cations or chemokines), whereas the binding to D1 does not [18].

Although the interaction of VCAM-1 with VLA-4 is essential for immunity, over expression of any of them or both may lead to several diseases pathologies such as multiple sclerosis [19], rheumatoid arthritis [20], allogeneic graft rejection [21], delayed-type hypersensitivity reactions [22], and tumor metastasis [23–25]. Thus, quantifying the affinity of VCAM-1/VLA-4 interaction is of prime importance to better understand its role in diseases as well as in developing the therapeutic drugs and strategies.

Quantifying the binding affinity of interacting protein pairs *in vitro* is fundamental in understanding these biochemical processes [26]. Among the several important parameters to quantify the binding affinity of protein-protein interactions, the equilibrium dissociation constant (K_d_) has been studied extensively through different techniques including isothermal titration calorimetry (ITC) [27], surface plasmon resonance (SPR) [28], and radio-ligand binding assay [29]. However, Förster/Fluorescence resonance energy transfer (FRET) [30] based protein-protein interaction assays provide many important advantages complementary to those of the other techniques. FRET assays are, in general, applicable both *in vitro* and *in vivo* [31, 32]. Moreover, FRET based measurements require only a general-purpose fluorescence spectrometer and/or microscope as compared to other methods which often require a much more complicated/sophisticated experimental platform. Besides, FRET based protein interaction assays do not require any special conjugation or orientation of the proteins on other surfaces, except conjugating the proteins with fluorophores. Hence, several efforts have been made to establish quantitative FRET based protein interaction assays [33, 34].

FRET is a distant-dependent process where, through dipole-dipole interactions, an excited fluorophore molecule (donor) transfers energy non-radiatively to another molecule (acceptor) [30, 31]. As the FRET efficiency is proportional to the inverse of the sixth power of the distance between the donor and acceptor, the FRET emission signal provides a high degree of spatial sensitivity (in the range of approximately 1 to 10 nm) as well as signal specificity; hence, FRET has become one of the most important and popular tools in biomolecular analysis [31]. However, using FRET efficiency to quantitatively interpret protein-protein interactions had encountered some major challenges as this parameter depends on several factors such as concentrations of the donor and acceptor molecules, the dissociation constant (K_d_) of the interacting protein pairs, and the intrinsic FRET between the acceptor/donor pair [35]. Moreover, another obstacle in the development of quantitative FRET to obtain K_d_ was to extract the FRET emission signal at the maximum acceptor emission wavelength from the mixed FRET emission spectrum which contains (i) the unquenched direct donor emission, (ii) the direct acceptor emission, and (iii) the actual FRET emission signal [36]. Song et al. [36–38] have recently reported the development of a theoretical and experimental protocol to obtain (K_d_) of the SUMO1/Ubc9 interaction. The major highlight of their work is the identification and the subsequent elimination of donor and acceptor emissions at the acceptor emission wavelength to extract the FRET emission signal from one single assay [36].

In this work, we present the development of an ‘*in solution*’ steady-state quantitative FRET assay using the organic fluorophore FRET pair of Alexa Fluor 488 (AF488) and Alexa Fluor 546 (AF546) to obtain the K_d_ of the VCAM-1/VLA-4 interactions using standard fluorescence spectroscopy technique; the conjugate Alexa Fluor 488-VLA-4 (AF488-VLA-4) was used as the FRET donor, and the Alexa Fluor 546-VCAM-1 (AF546-VCAM-1) as the acceptor. All the experiments were conducted in 96-well plate format. From our analysis of the FRET assay, the dissociation constant (K_d_) of the interaction between VCAM-1 and VLA-4 was determined to be 41.82 ± 2.36 nM. To further validate this value obtained from our FRET assay, we also measured K_d_ via SPR experiment and analysis; the two results (K_d_ = 41.82 ± 2.36 nM from FRET and 39.60 ± 1.78 nM from SPR) are in good agreement. To the best of our knowledge, no methods have been reported in literature to obtain K_d_ of the VCAM-1/VLA-4 interaction. This information and our method will be useful for drug screening/assessment based on the inhibition efficiency of the drug candidates to VCAM-1/VLA-4 interaction. In general, our assay can be adapted for applications in drugs development and screening associated with many other disease-related protein-protein interactions.

## Results and Discussion

**Fig 1:**
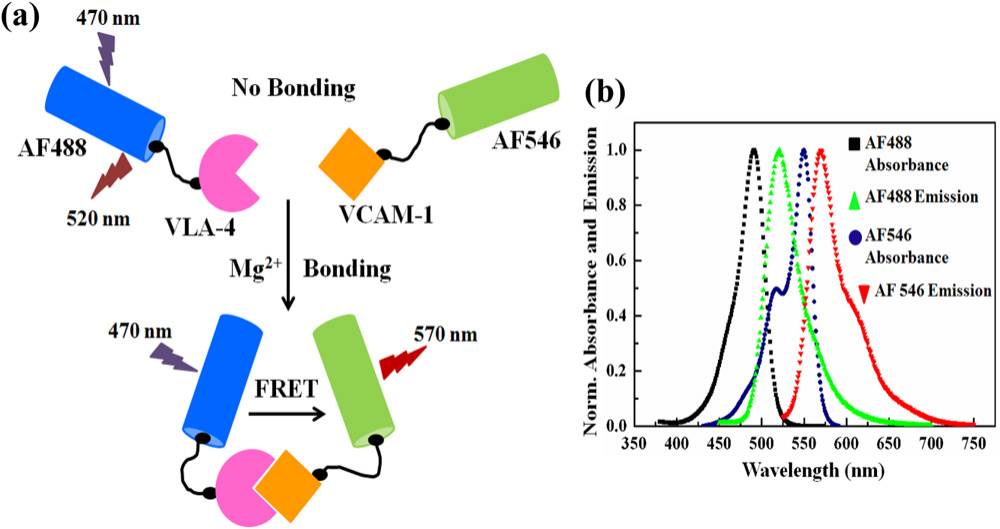
The FRET assay. (a) A schematic illustration of FRET with AF488-VLA-4 as the donor, and AF546-VCAM-1 as the acceptor conjugates. Upon excitation at 470 nm, two emission peaks, one at 520 nm, and the other at 570 nm were observed; the former originates from the free (unbounded) AF488-VLA-4, while the latter is due to FRET from the binding of the two protein conjugates. Proper amount of Mg^2+^ was added to the FRET mixture to induce conformational change of VLA-4 to enhance the binding affinity of VLA-4 and VCAM-1. (b) Normalized absorbance and emission spectra of AF488 and AF546 in PBS, pH 7.4.

In developing our quantitative “*in solution*” spectral FRET assay, we have used the dye-protein conjugates of AF488-VLA-4 as the donor and AF546-VCAM-1 as the acceptor. A schematic illustration of our FRET assay is shown in Fig. 1(a). The details of the FRET assay can be found elsewhere [39] with slight modification. Briefly, when there is no binding between VLA-4 and VCAM-1, only one emission peak at 520 nm (i.e., emission maximum of AF488) will appear when the mixture of protein conjugates were excited at 470 nm (the excitation maximum of AF488). In contrast, if the same FRET mixture is excited with 470 nm after addition of 2mM of MgCl_2_, which induces high-affinity conformational changes of VLA-4 and facilitates the interaction between VLA-4 and VCAM-1, a second emission peak at 570 nm (emission maximum of AF546) will also appear due to FRET between AF488-VLA-1 and AF546-VCAM-1. To confirm the fluorescence spectral characteristics of AF488 and AF546 in our experimental conditions, absorbance and emission spectra of these dyes were also recorded [Fig. 1(b)].

### Calculation of dye-to-protein (F/P) ratio in the conjugates

**Fig 2:**
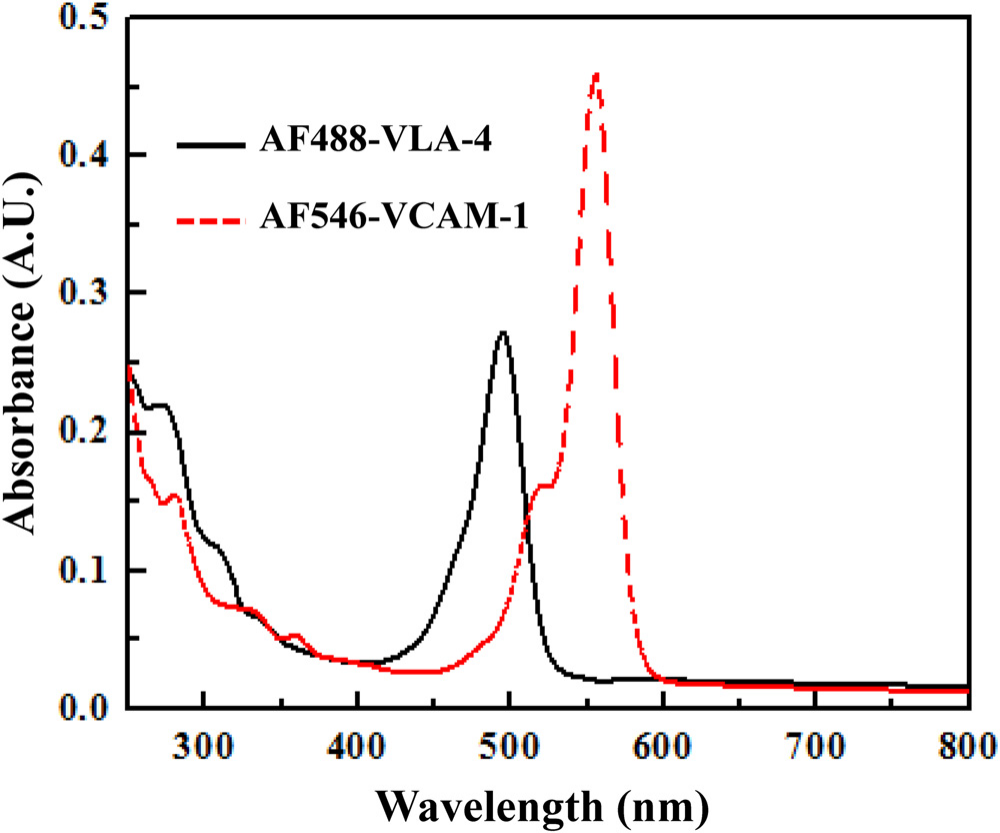
UV-visible absorption spectra of the dye-protein conjugate to obtain the dye-to-protein (F/P) molar ratio. The F/P ratios of AF488-VLA-4 and AF546-VCAM-1 were determined to be 3.0 ± 0.8 and 0.60 ± 0.02, respectively. These values were used to deduce the concentration of acceptors and donors in the dye-protein conjugate solutions. Each experiment was repeated three times under identical condition and the results are shown as arithmetic mean ± standard deviation.

In our dye-protein conjugates, AF488 and AF546 are amine reactive dyes which bind with non-protonated aliphatic amine groups, including the amine terminus of proteins and the ε-amino groups of lysines. The dye-to-protein (F/P) ratio, defined as the ratio of moles of fluorophores to moles of protein in the conjugate, specifies the degree of labelling of the proteins by the dyes. Here, the F/P ratios were obtained for both conjugates, AF488-VLA-4 and AF546-VCAM-1, from the UV-visible absorption spectra [Fig. 2], in conjunction with Equation (1) given below.

$$\frac{F}{P}=\frac{A_{\max}\times Dilution\;Factor}{\varepsilon_{dye}\times \left[p\right]}$$ (1)

where, A_max_ is the absorbance of the dye-protein conjugate at the dye absorption maxima, ε_dye_ is molar extinction coefficient of the dye, and [p] denotes the molarity of the protein. The F/P ratios of AF488-VLA-4 and AF546-VCAM-1 were determined to be 3.0 ± 0.8 and 0.60 ± 0.02, respectively.

The knowledge of F/P ratio is essential in developing FRET assays as this has significant impact on FRET activity. Too high labelling of proteins will give rise to prominent background signal whereas too low labelling poses the problem of isolating the FRET signal from background noise. Moreover, high F/P may result in concentration dependent quenching, which compromises on the sensitivity. However, the resultant FRET signal is determined by the acceptor-to-donor fluorophore ratio in the FRET mixture. The acceptor-to-donor fluorophore ratio is critical for fluorescence energy transfer [30, 40] which can be obtained from the F/P ratio and the amount of conjugated proteins in the mixture. Due to these reasons, we optimized our conjugation protocol to find an appropriate F/P ratio which provided significant FRET activity for subsequent development of our quantitative FRET assay.

### Observing the FRET emission

**Fig 3:**
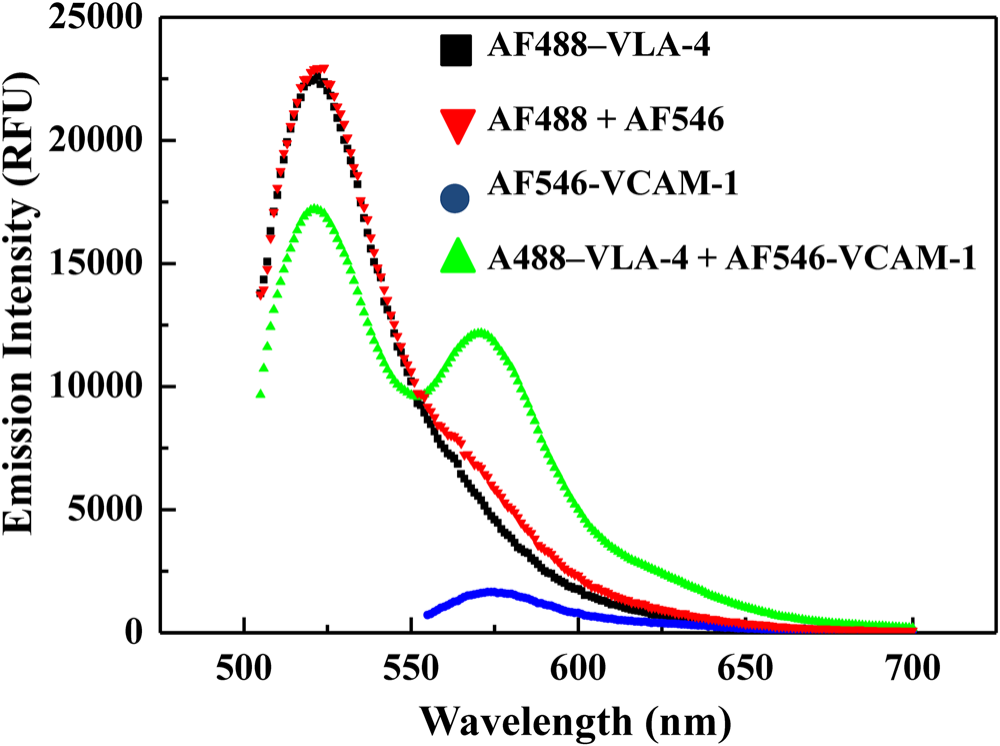
Confirming the FRET emission. The fluorescence emission spectra of AF488-VLA-4 (100 nM), AF546-VCAM-1 (100 nM), the mixture of AF488-VLA-4 (100 nM) and AF546 (100 nM), and the dyes only mixture of AF488 and AF 546 are compared. The excitation wavelength for the all the cases was 470 nm, the donor excitation maxima. The fluorescence spectrometer gain and integration time (20 μs) was kept constant while obtaining these spectra. For the mixture of A488-VLA-4 + AF546-VCAM-1, an acceptor sensitized emission was observed at 570 nm as compared to the AF488 + AF546 spectrum, which confirmed the FRET activity in our assay.

In the process of developing the quantitative FRET assay, we compared the fluorescence emission spectra of AF488-VLA-4 (100 nM), AF546-VCAM-1 (100 nM), the equimolar mixture of AF488-VLA-4 (100 nM) and AF546-VCAM-1 (100 nM), as well as similar equimolar mixture (corresponding to the dye concentrations in the dye-protein conjugates) of AF488 and AF546 dyes only to confirm the FRET activity in our assay under the excitation of 470 nm. The fluorescence spectra (Fig. 3) corresponding to these conditions indicate that the donor emission was quenched at 520 nm while the corresponding sensitized acceptor emission of the dye-protein conjugate mixture appeared at 570 nm. For the dyes only mixture, no additional peak around 570 nm appeared. These observations ascertained the FRET activity, and also indicated that the perturbation due to random FRET was negligible in our assay. These data also confirm the signal specificity of our FRET signal. Here, and in the rest of the article, the concentrations refer to the protein concentrations in the dye-protein conjugate, unless otherwise specified. From Fig. 3, it is also observed that the relative intensity of direct emission of AF546-VCAM-1 at 470 nm is significantly lower than that of the other cases, and may have little impact on the overall FRET emission signal. Rather than ignoring this weak signal, we did take it into account in quantifying our FRET emission signal, as can be found in the next section.

### Determination of the dissociation constant (K_d_) through FRET

**Fig 4:**
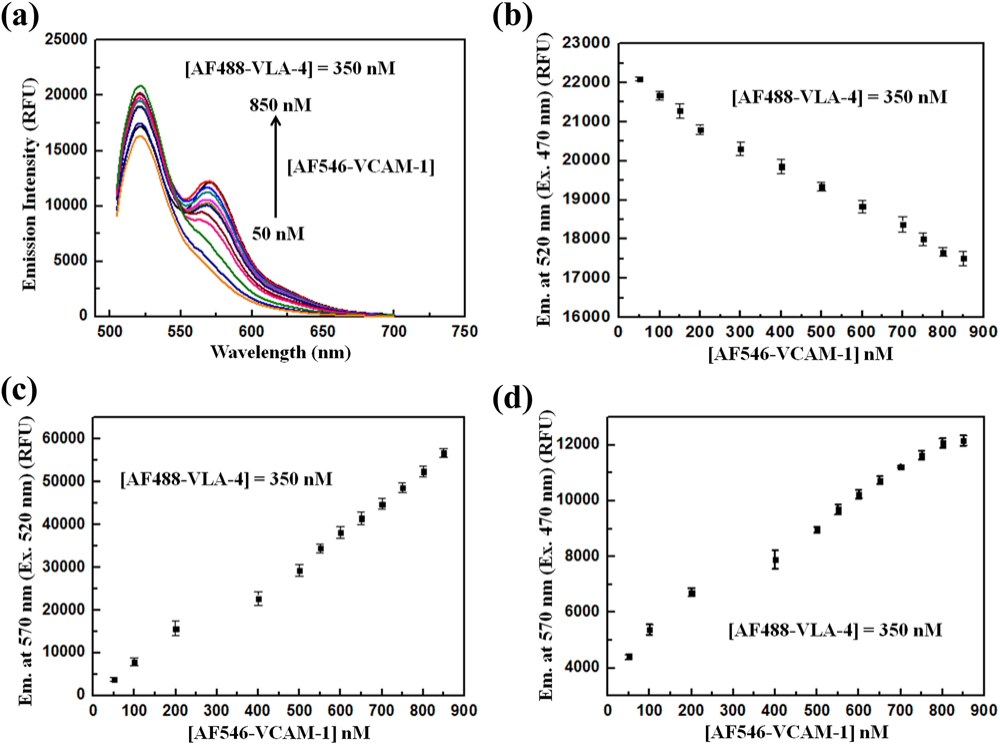
Determining the FRET signal at the acceptor emission maximum wavelength 570 nm. (a) Fluorescence emission spectra of the FRET mixtures (AF488-VLA-4 + AF546-VCAM-1). The FRET mixtures were excited at 470 nm. The concentration of AF488-VLA-4 was kept constant at 350 nM while that of AF546-VCAM-1 was varied from 50 nM to 850 nM. The various emission contributions at 570 nm of the FRET emission spectrum were obtained from these spectra at each AF546-VCAM-1 concentration. (b) The fluorescence emission signals of AF488-VLA-4 (D_emission_) at 520 nm in the FRET mixture upon 470 nm excitation. (c) Fluorescence emission signal of AF546-VCAM-1 (A_emission_) in the FRET mixture at 530 nm excitation. (d) The plot of total fluorescence emission of the FRET mixture (DA_emission_) at 570 nm with 470 nm excitation. Each measurement was repeated three times under identical condition to obtain the mean value and the standard deviation (indicated by the error bar).

In our steady-state FRET based assay to obtain the equilibrium dissociation constant (K_d_) of the VLA-4/VCAM-1 interactions, several concentrations of the acceptor conjugate, AF546-VCAM-1 (50–850 nM), were titrated to a fixed concentration of the donor conjugate AF488-VLA-4 (350 nM) to obtain the maximum FRET emission (maxFRET_emission_) signal, which in turn was used to obtain K_d_. The series of FRET emission spectrum corresponding to each AF546-VCAM-1 concentration, when excited at 470 nm, is shown in Fig. 4(a). From Fig. 4(a), we observe that each spectrum consists of two peaks, one at 520 nm (due to the unquenched emission of AF488-VLA-4, D_emission_) and other at 570 nm (DA_emission_). As was briefly described earlier in the “Materials and methods” section, the emission peak at 570 nm is composed of several components due to the cross-talk in emission detection as well as bleed-through in excitation. However, when the sample was excited at 520 nm, only one emission peak, (A_emission_), at 570 nm from AF546-VCAM-1, appeared. The absolute values of the donor emissions (D_emission_) at 520 nm for each concentration of AF546-VCAM-1 were shown in Fig. 4(b) when the FRET mixtures were excited at 470 nm which shows a steady quenching of the donor as the acceptor concentration increases, endorsing high FRET activity in our FRET assay. Moreover, the corresponding absolute acceptor emission values (A_emission_), under same conditions, when the FRET mixture was excited at 520 nm were also obtained as shown in Fig. 4(c). The total emission signal at 570 nm (DA_emission_) when the FRET mixture was excited at 470 nm is shown in Fig. 4(d) which also shows an increasing trend with increasing acceptor concentrations. The values of D_emission_, A_emission_, and DA_emission_ were used to calculate the FRET emission signal (FRET_emission_) for each acceptor concentration.

For any accurate and reproducible development of quantitative FRET assay, we need to take into account both the bleed-through in excitation and the cross-talk in emission detection, i.e. when the direct emissions of donor as well as acceptors also contribute to the FRET signal. Thus to obtain FRET_emission_ for each spectrum at 570 nm as shown in Fig. 3(a), the quantification of direct donor and acceptor emission contribution is required.

**Fig 5:**
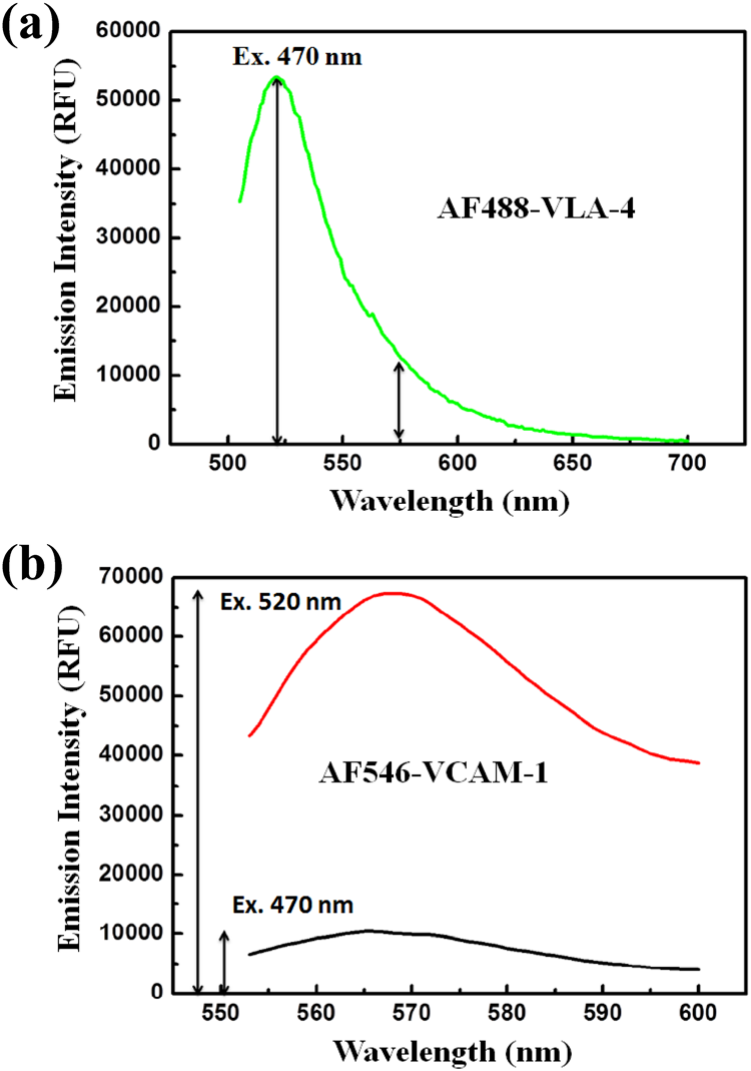
Determining the “μ” and “η”. (a) Fluorescence emission spectrum of AF488-VLA-4 (350 nM) alone when excited at 470 nm. The ratio constant “μ”, defined as the ratio of the emission signal at 570 nm to that at 520 nm of the AF488-VLA-4 emission spectrum was determined to be 0.128 ± 0.031. (b) Fluorescence emission spectra of AF546-VCAM-1 (850 nM) alone when excited at 470 and 520 nm. The ratio constant “η”, defined as the ratio of emission signal at 570 nm upon excitation at 470 nm to that at 570 nm when excited at 520 nm, was determined to be 0.143 ± 0.058. All the experiments were done in triplicate. The results are shown as arithmetic mean ± standard deviation.

Due to these facts, we assumed that the direct emission signal at 570 nm of AF488-VLA-4 is proportional to its maximum emission at 520 nm upon excitation at 470 nm. This proportionality constant, representing the ratio of the emission intensity of AF488-VLA-4 alone at 570 nm to that at 520 nm [Fig. 5(a)], denoted as “μ”, was determined to be 0.128 ± 0.031. Likewise, the ratio of the emission intensity of AF546-VCAM-1alone at 570 nm when excited at 470 nm to that at 570 nm when excited at 520 nm [Fig. 5(b)], denoted as “η”, was determined to be 0.143 ± 0.058. Now, multiplying “μ” with D_emission_ and “η” with A_emission_ give the direct emission contributions of the donors and acceptors respectively at 570 nm when the FRET mixture is excited at 470 nm; these results enable us to minimize the cross-talk effect in our FRET signal estimation. The resultant FRET_emission_ signal was obtained from the following relation involving the ratio constants as defined above,

$$FRET_{emission}=DA_{emission}-\mu \left(D_{emission}\right)-\eta \left(A_{emission}\right)$$ (2)

**Fig 6:**
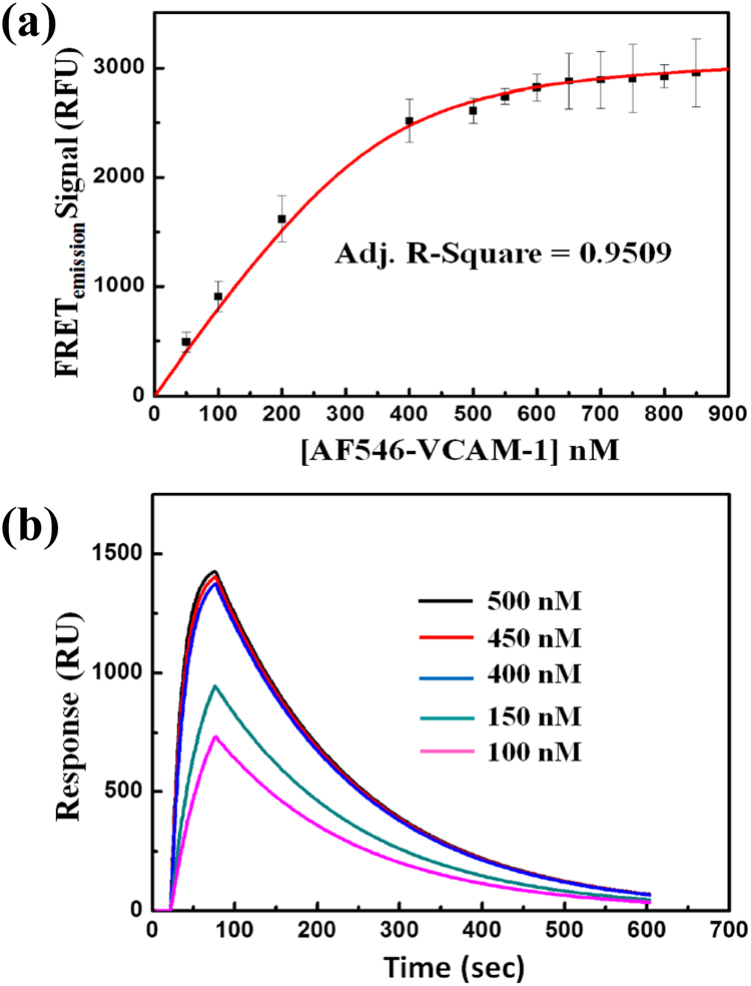
Determination of Kd through quantitative FRET and SPR. (a) Curve fitting of the experimental data representing the absolute FRET emission signals (FRET_emission_) with Equation (3). Each experiment was repeated three times under identical condition, and the error bar indicates the standard deviation; the results are shown as arithmetic mean ± standard deviation. The maximum FRET emission signal (maxFRET_emission_) and the corresponding equilibrium dissociation constant (K_d_) of the VLA-4/VCAM-1 interaction were determined to be 3184 RFU and 41.82 ± 2.36 nM, respectively. (b) Sensorgrams from the SPR sensor chip in BIAcore indicating the interaction of VCAM-1 (immobilized on the surface of the chip; details can be found in the text) with different concentrations (100, 150, 400, 450, to 500 nM) of VLA-4. The equilibrium dissociation constant (K_d_) of the VLA-4/VCAM-1 interaction was determined to be 39.60 ± 1.78 nM.

The absolute FRET_emission_ values, obtained from Equation (2), were further utilized to deduce the maxFRET_emission_ signal and subsequently K_d_ of the VLA-4/VCAM-1 interactions. Song et al. [37] have developed a systematic algorithm relating this FRET_emission_ and K_d_. Following this development and considering one-to-one interaction between the donors and acceptor conjugates, we obtained the value of K_d_ of our interaction of interest using the following relation [37],

$$FRET_{emission}=\text{max}FRET_{emission}\left[\frac{A-D-K_{d}+\sqrt{{\left(A-D-K_{d}\right)}^{2}+4K_{d}A}}{A-D+K_{d}+\sqrt{{\left(A-D-K_{d}\right)}^{2}+4K_{d}A}}\right]$$ (3)

where “A” and “D” denote the concentrations of AF546-VCAM-1 and AF488-VLA-4, respectively. The values of maxFRET_emission_ and K_d_ were obtained by fitting Equation (3), derived from non-linear regression analysis, with the datasets FRET_emission_ vs. AF546-VCAM-1 concentrations [Fig. 6(a)]. From our calculations, the estimated value of maxFRETemission was 3184 RFU and the equilibrium dissociation constant, K_d_, was 41.82 ± 2.36 nM.

### Determination of K_d_ via Surface Plasmon Resonance (SPR)

To further confirm our estimation of K_d_ of the VLA-4/VCAM-1 interaction, we employed surface plasmon resonance (SPR) based technique, another commonly used methodology to study protein-protein interactions, to determine the value of K_d_. In the SPR approach, the VCAM-1Fc was first immobilized on the sensor chip and several different concentrations of VLA-4 (100, 150, 400, 450, and 500 nM) were flowed in sequentially, one at a time, into the chamber to obtain corresponding binding response sensorgrams (details in “Materials and methods” section) as shown in Fig. 6(b). The value of K_d_, obtained via BIAcore analysis software using Langmuir model fitting [41], was 39.60 ± 1.78 nM. The consistency of the value of K_d_ obtained by these two different approaches (i.e., 41.82 ± 2.36 nM by FRET, and 39.60 ± 1.78 nM by SPR) serves to cross-check and validate each other, despite the fact that in the case of SPR, the protein interaction was measured without any fluorophore-conjugation.

## Conclusions

In this article, we report the successful application of “*in solution*” FRET based quantitative assay to obtain the equilibrium dissociation constant, K_d_, of the interaction between VLA-4 and VCAM-1. The assay is simple, robust, and easy to reproduce. The feasibility of the “*in solution*” quantitative FRET assay has often been challenged based on the argument that the in-solution tests may bear an inherent background signal due to contact between free-floating receptors as a result of unavoidable perturbations such as the Brownian motion and the microthermal currents in the sample holder. Hence, we applied another method, namely the surface plasmon resonance (SPR), where one of the proteins (in our case, VCAM-1Fc) is immobilized on a solid substrate and the other (VLA-4) is flowed into the chamber to monitor their dynamic interaction in real-time via the SPR signal. The values of K_d_ deduced from both techniques (41.82 ± 2.36 nM by FRET, and 39.60 ± 1.78 nM by SPR) are in excellent agreement. Our results confirm that the interaction between VLA-4 and VCAM-1 has a high affinity which forms the basis of a firm adhesion in leukocyte trafficking. To the best of our knowledge, this is the first to report the value of K_d_ of the VCAM-1/VLA-4 interaction, as well as the consistency in the values (of K_d_) determined by two different approaches, in-solution with dye-conjugate in the case of FRET vs. without dye-conjugate and with one of the proteins immobilized on a solid substrate in the case of SPR. This information may serve as a reference point for drug screening based on the inhibition efficiency to VCAM-1/VLA-4 interaction of the drug candidates. In general, our assay can be adapted for applications in drugs development and assessment associated with many other disease-related protein-protein interactions.

## Materials and Methods

### Materials

The proteins, human recombinant VCAM-1, human recombinant VCAM-1 Fc chimera and human recombinant VLA-4, were purchased from R&D systems (Minneapolis, USA). These soluble forms of proteins have already been extensively used in several studies [42–45], which demonstrate the feasibility of using them to develop in-solution assays. The conformational state of the soluble form of the recombinant VLA-4 from R&D systems has not been well defined; however, consistent observation of the biological activity of the purified protein indicates that the protein is properly folded to bind with VCAM-1 with consistent affinity [42, 43]. The dyes, Alexa Fluor 488 carboxylic acid, succinimidyl ester and Alexa Fluor 546 carboxylic acid, succinimidyl ester, were procured from Molecular Probes (Eugene, Oregon, USA). The organic solvent, dimethyl sulfoxide (DMSO) dried, was obtained from Merck (Darmstadt, Germany). The dye-protein conjugates were purified through Slide-A-Lyzer dialysis cassette (0.5 ml, 10K MWCO) from Thermo Scientific (Rockford, USA). The FRET experiments were performed in low autofluorescence transparent flat 96 well plates (BD Biosciences, Bedford, MA, USA). The chemicals, N-(3-Dimethylaminopropyl)-N′-ethylcarbodiimide (EDC), n-hydroxysuccinimide (NHS), and ethylene glycol-bis (2-aminoethylether)-N, N, N′, N′-tetraacetic acid (EGTA), were from Sigma-Aldrich, Saint-Louis, USA. For the surface plasmon resonance (SPR) experiments, Sensor chip CM5 (BR-1000–12) and human antibody capture kit for VCAM-1 immobilization (BR-1008–39) were obtained from GE Healthcare, Little Chalfont, UK.

### Methods

#### Conjugation of dyes to proteins and purification

The FRET dye pair of AF488 and AF546 was conjugated to VLA-4 and VCAM-1, respectively, following the protocol modified from Molecular probes labelling kits. In short, for each case the protein was dissolved in 50 μl sodium bicarbonate buffer, pH 9.0, at a concentration of 1mg/ml. The dye solution was prepared in DMSO at 1mg/ml and used immediately. 2 μl of dye solution was added to the 50 μl protein solution and was kept for 2 hours in the dark at room temperature. The free dyes, after the completion of the conjugation reaction, were removed by exhaustive dialysis using Slide-A-lyzer dialysis cassette G2 against phosphate saline buffer (PBS), pH 7.4, for 18 hours at 4°C. Subsequently, the protein concentrations as well as dye to protein (F/P) molar ratios were obtained for each case, i.e., AF488-VLA-4 and AF546-VCAM-1, by UV-visible absorbance measurements via a commercial spectrophotometer (DU-800, Beckman Coulter, Fullerton, Germany).

#### FRET spectrum analysis for dissociation constant (K_d_) determination

For the FRET mixture, AF488-VLA-4 and AF546-VCAM-1 conjugates were diluted in PBS buffer containing 1mM EGTA, 2 mM magnesium chloride (MgCl_2_), pH 7.4 to a total volume of 50 μl and was incubated for 30 minutes. The final concentration of AF488-VLA-4 was maintained at 350 nM while that of AF546-VCAM-1 was varied from 0 to 850 nM. The concentration values, here and in the rest of the article, correspond to the protein concentration in the dye-protein conjugate solutions and not to the dye concentration unless otherwise stated. The FRET mixtures were transferred into 96-well plates and FRET emission spectrum for each well was recorded by a fluorescence multi-well plate reader, Infinite M200 pro (Tecan, Grödig, Austria). The emission at 520 and 570 nm were measured at the donor excitation wavelength of 470 nm; moreover the emission at 570 nm was also measured at the acceptor excitation maximum of 520 nm. The background corrected fluorescence emission signals were obtained by averaging over three experiments with each sample at each specific condition (such that each datum point represents the mean value of 9 measurements with 3 samples).

VLA-4 shows several affinity states depending on the presence of single specific divalent cations or combinations of several divalent cations [46]. In our study, we followed the experimental protocol suggested by Masumoto et al. [47] which showed that individual divalent cations (Mg^2+^, Mn^2+^, or Ca^2+^) suffice to induce affinity state changes of VLA-4 in interacting with VCAM-1. Specifically, we used only Mg^2+^ (by adding MgCl_2_ in the FRET mixture) as the primary agent to induce affinity changes in VLA-4. Under such condition, the interaction affinity of VLA-4 and VCAM-1 in solution is mainly regulated by the amount of divalent cations [42, 45, 48], in contrast to the complex environment in case of cell-cell contacts.

When the FRET mixture of AF488-VLA-4 and AF546-VCAM-1 was excited at 470 nm, two emission peaks, one at 520nm, and the other at 570 nm, appeared in the emission spectrum. The emission peak at 520 nm was due to the unquenched emission of AF488-VLA-4 (D_emission_). However, the emission peak at 570 nm (DA_emission_) was consisted of (1) unquenched emission of AF488-VLA-4; (2) direct emission of AF546-VCAM-1; and (3) the emission of AF546-VCAM-1 due to the non-radiative energy transfer from AF488-VLA-4 (FRET_emission_). When the same FRET mixture was excited at 520 nm, only one emission peak at 570 nm (A_emission_), due to the direct emission of AF546-VCAM-1, was observed. By subtracting the unquenched donor and acceptor emission contributions from the total emission at 570 nm (at 470 nm excitation), the FRET emission signal was obtained and further utilized to obtain the dissociation constant (K_d_) of the interaction between VCAM-1/VLA-4; more detail is given in Section 3.2.

#### Surface plasmon resonance (SPR) to obtain the K_d_

To further validate the FRET based estimation of K_d_, the VCAM-1/VLA-4 interaction was also studied by SPR. The experiments were conducted via BIAcore 3000 CM5 sensor chips (BIAcore International SA, Neuchâtel, Switzerland) with carboxymethylated dextran covalently attached on the gold surface. The flow rate was fixed at 5 μl/min. The carboxyl acid functional groups on the sensor chip surface were activated by 400 mM EDC/100 mM NHS for 7 minutes, and 25 μg/ml anti-human IgG (Fc) was immobilized on the sensor surface by flowing into the chamber channel. This step was necessary to maintain the orientation of the VCAM-1Fc on the sensor surface as the anti-human IgG (Fc) binds only with the Fc region of the VCAM-1Fc. Subsequently, 40 μg/ml of VCAM-1Fc in PBS, containing 1mM EGTA and 2mM Mg^2+^, was flowed into the channel for immobilization on the sensor surface; VLA-4 with a concentration 100 nm was then flowed into the channel and the SPR response curve was recorded. To obtain the K_d_ of VCAM-1/VLA-1 interaction, the experiment was repeated with different concentrations of VLA-4 (150, 400, 450, and 500 nM). After the completion of each experiment corresponding to one specific concentration of VLA-4, the sensor surface was regenerated by the regeneration buffer (from the human antibody capture kit; 3 M magnesium chloride) for subsequent usages with other concentrations of VLA-4. The dissociation constant, K_d_, was then obtained via BIAcore analysis software (BIAevaluation 3.2 RC1) by averaging over 3 experimental curves (i.e., repeating the same experiment three times) for each concentration.
